# Recurrent nontraumatic common peroneal neuropathy after surgical decompression: Case report and literature review

**DOI:** 10.1097/MD.0000000000045899

**Published:** 2025-11-21

**Authors:** Yi‐Hsuan Lee, Yi-Chan Lee, Meng-Chao Peng, Chung-Pei Chen, Ing-Ho Chene

**Affiliations:** a Department of Orthopedics, Taipei Tzu Chi Hospital, Buddhist Tzu Chi Medical Foundation, New Taipei City, Taiwan; b Department of Otolaryngology, Chang Gung Memorial Hospital, Keelung, Taiwan; c Department of Surgery, Sijhih Cathay General Hospital, New Taipei City, Taiwan; d Department of Orthopedics, Hualien Tzu Chi Hospital, Buddhist Tzu Chi Medical Foundation, Hualien, Taiwan.

**Keywords:** CPN, nontraumatic, peroneal nerve decompression, recurrence

## Abstract

**Rationale:**

Common peroneal neuropathy (CPN) is a common peripheral neuropathy usually caused by trauma or prolonged compression. Nontraumatic and recurrent cases, however, are rare, especially in older adults. This report presents a case of recurrent nontraumatic CPN, emphasizing the diagnostic and therapeutic challenges.

**Patient concerns:**

A 69-year-old male presented with sudden right-sided foot drop, numbness, and paresthesia without recent trauma.

**Diagnoses:**

Magnetic resonance imaging and electrodiagnostic studies confirmed nontraumatic CPN with compression near the fibular head.

**Interventions:**

The patient underwent surgical decompression and neurolysis of the common peroneal nerve, resulting in initial recovery. Two years later, symptom recurrence prompted re-evaluation, and a second decompression surgery was performed due to adhesions and scar tissue formation.

**Outcomes:**

The patient achieved complete sensory and motor recovery within 3 months after the second surgery, with no postoperative complications.

**Lessons:**

Recurrent nontraumatic CPN may result from postoperative adhesions or incomplete decompression, particularly in older adults. Comprehensive diagnostic workup, including magnetic resonance imaging and electrodiagnostic studies, and extended decompression with careful follow-up are essential to prevent recurrence and ensure optimal recovery.

## 1. Background

The common peroneal nerve, known as the common fibular nerve, is a branch of the sciatic nerve, plays a vital role in ankle dorsiflexion and the sensation of the lower extremities.^[1]^ Common peroneal neuropathy (CPN), the most common peripheral neuropathy affecting the lower limbs, may stem from traumatic and nontraumatic causes^[2–4]^ and results in several degrees of muscle weakness with symptoms such as foot drop, foot dragging, weakness, and numbness. Traumatic causes may include fractures, knee dislocations, sprains, direct impact injuries, or prolonged compression due to casts or immobilization around the knee. Nontraumatic causes can encompass prolonged external pressure on the nerve (such as from habitual leg crossing or prolonged squatting), metabolic conditions (e.g., diabetes), mass lesions (e.g., cysts, tumors, or ganglions), and inflammatory or degenerative disorders affecting the nerve. Each of these factors may produce nerve compression, stretching, and ischemia that lead to demyelination or even axonal damage, resulting in varying degrees of functional impairment.^[5,6]^ However, some cases remain idiopathic, with no identifiable underlying cause.^[7]^ Early intervention, including conservative treatments such as physical therapy, splinting, and anti-inflammatory measures, often promotes favorable nerve recovery. Surgical decompression may be considered in specific cases, particularly for patients with persistent or severe symptoms, progressive deficits, or identifiable structural nerve compression, such as from trauma or a mass lesion, when conservative management has failed.^[8]^ Additionally, electrodiagnostic evidence showing significant axonal loss or conduction block may support the need for surgical intervention to prevent further nerve damage and improve functional outcomes.^[2–4]^

Here we present a case of nontraumatic peroneal nerve palsy that required decompression surgery; subsequently, the patient experienced recurrence, and underwent a second surgical intervention. This report includes a comprehensive overview of the onset, diagnostic process, and treatment approach, along with detailed magnetic resonance imaging (MRI) and neurophysiological assessments. Given the rarity of nontraumatic cases, and the even rarer recurrence, this case offers valuable insight to support and inform future clinical decision-making.

## 2. Case presentation

**Figure 1.**
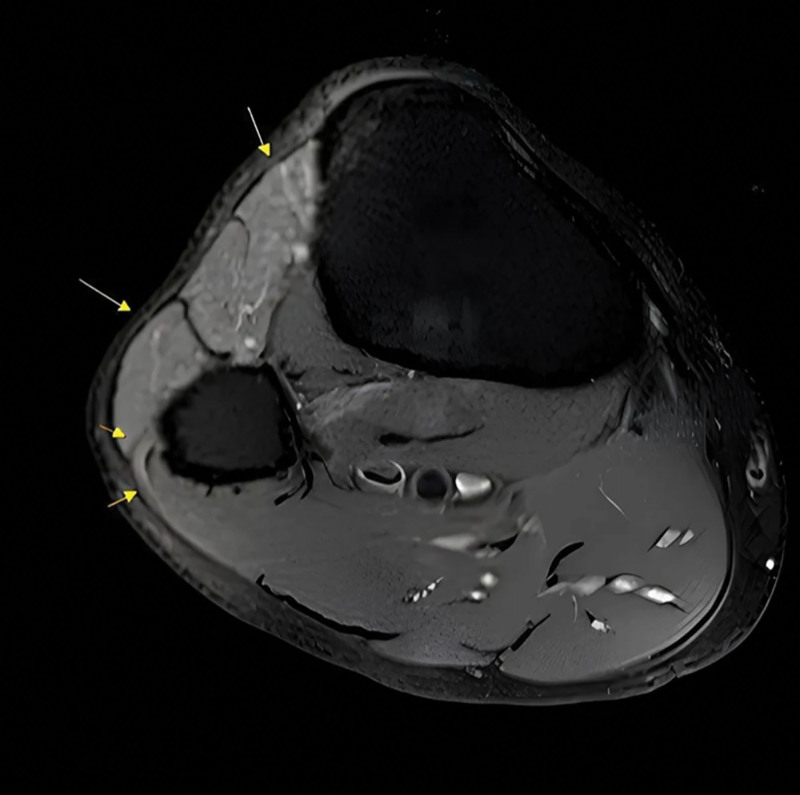
The short yellow arrow indicates the common peroneal nerve as it courses around the fibular head, showing increased T2-weighted (T2W) signal, which suggests nerve injury accompanied by inflammation and edema. The long yellow arrow indicates the anterior compartment muscle, with increased T2W signal, suggesting edema due to denervation. This depiction provides further evidence of injury to the common peroneal nerve, which innervates the affected muscle group.

In early January 2021, a 69-year-old male presented to the orthopedic outpatient clinic of our institution complaining of a sudden onset of right-sided foot drop, not related to any recent direct trauma, accompanied by numbness and paresthesia over the lateral leg and foot, without significant pain. He reported a 2-week history of frequent right ankle sprains and limping, which may have contributed to indirect nerve stress. He reported frequent right ankle sprains and limping over the past 2 weeks, without a history of direct trauma, systemic disease, or medication use. These sprains may have caused indirect mechanical stress on the nerve. Physical examination revealed muscle strength of BMRC grade 1 in dorsiflexion and toe extension, grade 2 in eversion, preserved ankle plantar flexion, and full active range of motion at the knee. Sensory examination showed reduced sensation over the lateral leg and dorsum of the foot. Tinel sign was positive at the fibular head. Under suspicion of lumbar spine pathology contributing to the foot drop, a lumbar MRI demonstrated a mild herniated intervertebral disc and degenerative disc changes at the L3–4 and L4–5 levels, without significant radicular compression. To further evaluate peripheral nerve involvement and exclude any distal pathology, a lower leg MRI was performed on the fourth day after symptom onset, revealing increased T2 signal intensity in the tibialis anterior muscle, suggestive of early denervation changes, along with hyperintensity of the common peroneal nerve at the level of the fibular head (Fig. 1). Nerve conduction velocity (NCV) and electromyography (EMG) demonstrated findings consistent with acute involvement of the right common peroneal nerve, supporting the clinical diagnosis.

**Figure 2.**
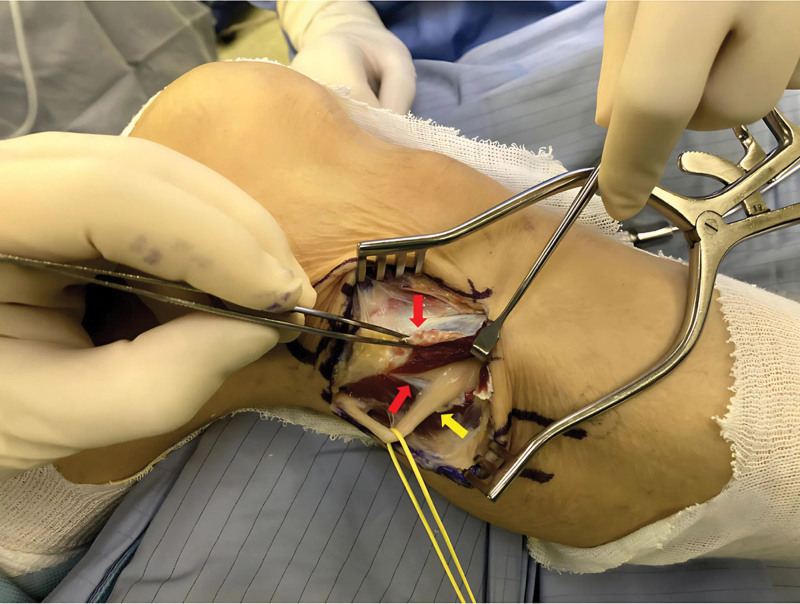
The red arrow indicates the incised lateral compartment fascia during surgery, which was identified as the primary site of nerve compression. The nerve appears flattened due to compression, and no accompanying microvasculature is observed on its surface (yellow arrow).

Surgical decompression and neurolysis of the right common peroneal nerve were arranged for the patient and were performed by our team approximately 1 month later. Intraoperatively, a tight muscle septum near the fibular head—identified as the primary compression site—was found to be causing significant pressure on the common peroneal nerve. Additionally, superficial compression by the overlying fascia and peroneus longus muscle may also have contributed to nerve entrapment, as illustrated in Figure 2. The development of a tight muscle septum may be related to chronic mechanical stress, localized hypertrophy, or anatomical predisposition, although the exact mechanism remains unclear.

Surgical decompression was performed through a lateral approach, with stepwise release of the intermuscular septum, superficial fascia, and overlying peroneus longus muscle. The common peroneal nerve was thoroughly decompressed both proximally and distally to the fibular head to ensure complete nerve release. After the decompression procedure, the patient reported an immediate 80% reduction in numbness and paresthesia, with muscle strength improving to grade 2. During regular follow-ups, the patient progressively recovered motor and sensory function, with complete functional restoration by 3 months postoperatively.

**Figure 3.**
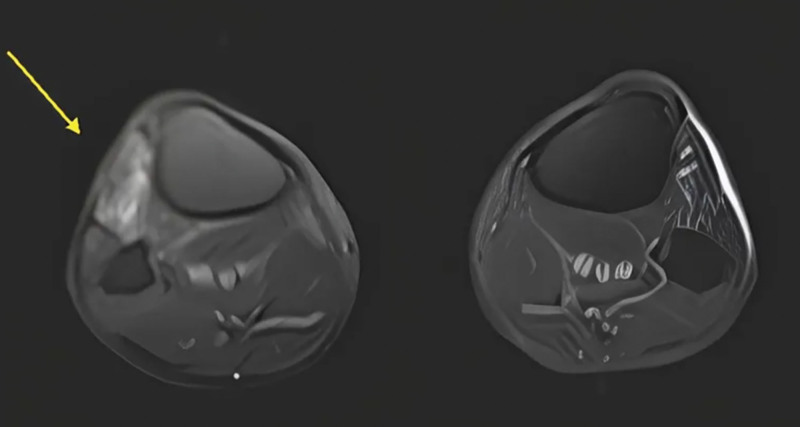
Anterior compartment muscle atrophy and edema due to denervation show the extent of injury to the common peroneal nerve, which innervates these muscles (yellow arrow).

**Figure 4.**
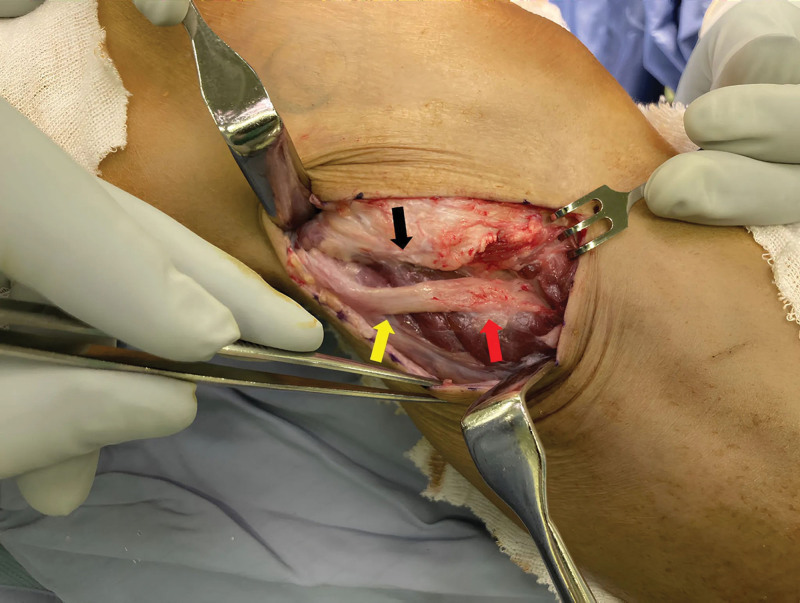
The yellow arrow indicates the site of nerve compression, appearing pale and bloodless, contrasting with the normal color of unaffected nerve tissue (red arrow). The black arrow indicates the site of nerve compression caused by adhesive tissues within the lateral compartment fascia, with the area posteriorly corresponding to the most prominent point of the fibular head.

However, after 2 years, the patient experienced a recurrence of symptoms, including foot drop, numbness, and paresthesia, and returned to the outpatient department 2 years later. Lower leg MRI demonstrated persistent T2 hyperintensity of the common peroneal nerve at the fibular head, along with high signal changes in the anterior and lateral muscle compartments and visible atrophy in the anterior compartment muscles, suggestive of chronic denervation. These findings appeared consistent with, rather than new compared to, the initial presentation (Fig. 3). Right common peroneal nerve decompression was administered by our team 5 days later, through the previous operative wound. Intraoperatively, dense scar tissue and fibrous adhesions were observed surrounding the common peroneal nerve at the level of the fibular neck and extending proximally along the intermuscular septum. The nerve was fully decompressed both above and below the fibular head, with dissection extending approximately 6 cm proximally and distally to ensure complete release of any compressive areas (Fig. 4). After surgery, recovery was rapid, with complete resolution of numbness and paresthesia within 2 months and full muscle strength and functional recovery achieved by 3 months. NCV and EMG were performed prior to both surgical procedures. Postoperative studies were not repeated due to the patient’s rapid symptom resolution and full clinical recovery.

## 3. Discussion

This case report presents a 69-year-old male with common peroneal neuropathy likely related to repetitive mechanical stress from frequent ankle sprains, leading to foot drop, numbness, and paresthesia. After initial diagnosis, the patient underwent surgical decompression of the right common peroneal nerve, which led to significant symptom improvement and functional recovery and a tight muscle septum near the fibular head was found to be causing significant pressure on the common peroneal nerve. However, symptoms recurred 2 years later, prompting a second decompression surgery, during which dense scar tissue and adhesions were identified as the recurrent compressive etiology. Following the second procedure, the patient still experienced full recovery, with symptoms completely resolving within 3 months. The mechanistic pathway in this case likely reflects an interplay between preexisting structural susceptibility, repetitive microtrauma, and age-related healing characteristics. Frequent ankle sprains may cause chronic traction and shear stress on the peroneal nerve, predisposing to intraneural ischemia, localized edema, and subsequent fibrotic changes,^[9,10]^ while in older adults, reduced connective tissue elasticity and vascular supply further increase the risk of postoperative adhesion formation.^[11]^ To our knowledge, such sequential etiologies in an elderly patient without trauma—initial compression from a tight muscle septum followed by recurrent compression from postoperative adhesions—have rarely been documented in the literature, underscoring the clinical relevance and educational value of this report.

To summarize briefly, the present case report illustrates, in particular, that while nontraumatic peroneal nerve palsy can respond well to decompression surgery, cases may recur especially in older patients with structural nerve compression. MRI findings, along with electrodiagnostic testing, were essential in diagnosing the nerve involvement and guiding surgical decisions. Although recurrence is considered uncommon in peroneal nerve palsy, especially in atraumatic cases, it has been reported in the literature, often associated with incomplete decompression or postoperative adhesions.^[7]^ In this case, the recurrence may reflect residual or insufficient release during the initial surgery, particularly at the anterior or posterior aspects of the nerve, compounded by fibrotic adhesion at the prior operative site.^[12]^ This underscores the importance of a thorough surgical approach—extending both proximally above the fibular neck and distally beyond the fibular tunnel—to ensure complete relief of all potential compressive forces.^[13]^ Compared with conventional decompression, the extended decompression used in this case incorporated proximal release into the peroneus longus tunnel and distal release into the intermuscular septum, which may help reduce recurrence risk, especially in revision cases.^[12]^

While the present case report describes an older adult male aged 69 years with a recent history of frequent ankle sprains as a likely precipitating factor, and acute onset with timely intervention, he also presented with common symptoms (foot drop, numbness, and paresthesia) that recurred after initial successful treatment. Previously reported cases include a series of cases that consisted of younger patients with sudden onset of symptoms in some and subacute or chronic onset in others and a similar lack of trauma or precipitating factors.^[14]^ Another report describes a younger patient with sudden onset of nontraumatic CPN, with symptoms of right-sided foot drop but no precipitating factors. The authors suggest that in such cases with idiopathic foot drop, clinicians must consider causes such as CPN entrapment.^[15]^ High-energy injuries are rare in neurosurgery, while low-energy injuries with prolonged compression, such as entrapment, are more common.^[8]^ A recent review supports the notion that most cases of CPN result from low-energy mechanisms such as entrapment or prolonged compression. However, certain high-energy injuries—most notably knee dislocations—remain among the most common causes of traumatic peroneal nerve injury and should not be overlooked.^[16]^

Symptoms, of course, suggest the necessary diagnostic workup. For the present case, diagnosis leveraged the results of physical examination, MRI, and NCV and EMG. As noted above, MRI findings, along with electrodiagnostic testing, were essential in diagnosing the nerve involvement and guiding surgical decisions. In that report, Fortier et al^[8]^ emphasized that MRI findings, along with electrodiagnostic testing, were essential in diagnosing the nerve involvement and guiding surgical decisions. Vandenbergh et al^[17]^ also support this approach, emphasizing that peroneal nerve issues are rare and typically assessed through clinical and electromyographic means, but at the same time, they pointed out the usefulness of MRI to identify nerve lesions without specialized sequences. Recently, ultrasound has also emerged as a valuable tool in the evaluation of peripheral nerve pathologies, including CPN. It provides dynamic, real-time imaging, allows direct visualization of nerve continuity, and may be superior to MRI in detecting subtle entrapments or identifying surrounding soft tissue structures in specialized settings. Authors of another previous case series reviewed 11 consecutive patients (10 males, 1 female) 8 of whom presented with foot drop and 3 with sensory deficits. Diagnosis was confirmed in each case by MRI—which was also used effectively for monitoring during treatment. Based on causes of those cases, the authors suggested that ganglion cyst was the most common cause of nontraumatic CPN, followed by osteochondroma, synovial cyst and aneurysm, but not in a specific order.^[18]^ Another case report describes a unique case of a younger, otherwise healthy male (age 36 years) with sudden right foot drop (experienced while trying to stand from overnight bedrest) who was diagnosed as nontraumatic CPN based on results of nerve conduction studies and electromyography, which showed decreased amplitude of the right peroneal nerve at the knee and denervation of the tibialis anterior and extensor digitorum brevis, respectively.^[15]^ For adequate and accurate diagnosis, Yang et al^[16]^ stipulate that integrating MRI and energy-dispursive X-ray analysis may aid early and accurate identification of CPN injuries, which is crucial for timely intervention.

Walking, as we know, depends on the dorsiflexion function of the peroneal nerve. The deep peroneal nerve provides motor innervation to the tibialis anterior and toe extensors, enabling ankle dorsiflexion and toe extension, and supplies sensory input to the first interdigital web space. In contrast, the superficial peroneal nerve controls ankle eversion through the peroneus longus and brevis muscles and provides sensory innervation to the anterolateral lower leg and dorsum of the foot.^[18]^ Understanding these functions supports treatment choices along with underlying causes. Myers et al^[15]^ describe a unique case of entrapment of the peroneal nerve resulting in CPN, which was treated successfully. That case report describes a younger otherwise healthy male (age 36 years) with right-sided foot drop that appeared suddenly when simply trying to stand on walk in the morning. He was treated surgically after not responding to 5 days of prednisone, undergoing surgical release of the peroneal nerve and excision of the boney prominence. At 12 days post-op there was no sensory improvement but motor examination did reveal improvement. Although sensory improvement was slow the patient did achieve a return of normal activity within months.^[14]^ Microsurgical decompression was the treatment applied by Tarabay et al^[7]^ for a patient diagnosed with severe idiopathic common fibular nerve entrapment. The authors recommend microsurgical decompression in order to get the most favorable outcome. A case series of 15 patients with peroneal nerve entrapment reported 10 younger patients with idiopathic cases predominated by motor deficits. Following neurolysis, they achieved gradual recovery within 2 months and full sensory and motor function later. The remaining cases included 3 with common peroneal nerve injury from ankle trauma and 2 from postural compression.^[16]^ The primary concern in entrapment cases was the risk of persistent functional impairment, prompting early neurolysis as the treatment goal. A previous study reported a mean time to intervention of 7 months (range: 2–18 months), with a mean follow-up of 42 months (range: 25–62 months). Functional recovery was achieved within an average of 2.5 months (range: 2 weeks–6 months).^[19]^ This case underscores the clinical challenge of symptom recurrence following initial surgical success in common peroneal neuropathy. Despite an initially favorable outcome, the patient experienced delayed recurrence likely related to scar tissue formation. This highlights the importance of long-term follow-up and the need for surgeons to consider extended decompression and meticulous dissection during the first surgery to minimize future compression risks. The second surgical outcome remained good (no postoperative complications), reinforcing the effectiveness of thorough reexploration in recurrent cases. Extended decompression techniques may help to minimize these risks. Additionally, as described above, regular follow-up care is crucial to monitor for any recurrence of symptoms, allowing for timely intervention and improving patients’ long-term outcomes.

### 3.1. Future outlook

In reporting the present case, we have highlighted the use of and importance of a comprehensive diagnostic approach that relies on detailed imaging and electrodiagnostic testing essential for accurately identifying nerve damage and pinpointing compression sites. In treating the present case, detailed imaging, electrodiagnostic testing, and thorough intraoperative findings contributed valuable insight into this rare case of recurrent nontraumatic CPN. The patient’s history of frequent ankle sprains offers valuable context for understanding the etiology of nerve injury, highlighting the role of repetitive mechanical stress as a modifiable risk factor. Overall, because recurrent nontraumatic CPN is rare and clinician experience is obviously lacking, additional case reports and studies with sufficient data analysis are still needed in order to develop meaningful management strategies for these rare peroneal neuropathies. In particular, future research should focus on refining surgical techniques and identifying critical steps—such as the extent of decompression proximally and distally—to reduce the risk of incomplete release and recurrence. Nevertheless, the relatively short follow-up period after the second surgery limits definitive conclusions regarding long-term surgical efficacy, and longer-term observation is warranted. Lastly, we recommend that, in the future, a structured postoperative rehabilitation program emphasizing ankle dorsiflexor strengthening, proprioceptive training, and scar mobilization be further studied for its potential to reduce recurrence risk and optimize functional recovery.

## 4. Conclusions

The present case report underscores the complexities in managing recurrent nontraumatic CPN. Reporting this rare case has shown that surgical decompression provides substantial relief and functional recovery, as seen in the initial treatment outcome. However, the recurrence of symptoms highlights the potential for ongoing compressive forces, possibly due to scar tissue and adhesions, which may require further intervention. A comprehensive diagnostic workup, including MRI and electrodiagnostic studies, remains crucial in identifying the precise compressive factors and guiding effective surgical decompression. This case emphasizes the importance of extended decompression techniques to prevent recurrence, particularly in anatomically vulnerable areas. For clinicians, this report provides valuable insight into the necessity of thorough decompression and vigilant follow-up to optimize management and mitigate the risk of recurrence in nontraumatic CPN.

## Author contributions

**Conceptualization:** Yi-Hsuan Lee.

**Data curation:** Yi-Hsuan Lee, Ing-Ho Chene.

**Formal analysis:** Yi-Chan Lee, Ing-Ho Chene.

**Investigation:** Yi-Chan Lee, Meng-Chao Peng.

**Methodology:** Yi-Chan Lee, Meng-Chao Peng, Chung-Pei Chen.

**Project administration:** Yi-Hsuan Lee.

**Resources:** Yi-Chan Lee, Meng-Chao Peng, Chung-Pei Chen, Ing-Ho Chene.

**Software:** Meng-Chao Peng, Ing-Ho Chene.

**Supervision:** Yi-Hsuan Lee, Chung-Pei Chen.

**Visualization:** Yi-Hsuan Lee.

**Writing – original draft:** Chung-Pei Chen.

**Writing – review & editing:** Chung-Pei Chen.
